# Prognostic differences between oligometastatic and polymetastatic extensive disease-small cell lung cancer

**DOI:** 10.1371/journal.pone.0214599

**Published:** 2019-04-19

**Authors:** Masayuki Shirasawa, Tomoya Fukui, Seiichiro Kusuhara, Shinya Harada, Noriko Nishinarita, Yasuhiro Hiyoshi, Mikiko Ishihara, Masashi Kasajima, Satoshi Igawa, Masanori Yokoba, Hisashi Mitsufuji, Masaru Kubota, Masato Katagiri, Jiichiro Sasaki, Katsuhiko Naoki

**Affiliations:** 1 Department of Respiratory Medicine, Kitasato University School of Medicine, Kanagawa, Japan; 2 Department of Medical Laboratory, Kitasato University School of Allied Health Sciences, Kanagawa, Japan; 3 Fundamental Nursing, Kitasato University School of Nursing, Kanagawa, Japan; 4 Research and Development Center for New Medical Frontiers, Kitasato University School of Medicine, Kanagawa, Japan; University of South Alabama Mitchell Cancer Institute, UNITED STATES

## Abstract

**Purpose:**

Oligometastasis is a state in which cancer patients have a limited number of metastatic tumors; patients with oligometastases survive longer than those with polymetastases. Extensive disease (ED)-small cell lung cancer (SCLC) is considered a systemic disease and a poor survival. This study investigated whether the concept of oligometastases is prognostic factor also applicable to patients with ED-SCLC.

**Methods:**

We performed a retrospective study of 141 consecutive patients with ED-SCLC between 2008 and 2016. The patients were divided into four subgroups: group 1; patients with solitary metastatic site in one organ (n = 31), group 2; patients with 2–5 metastatic sites in one organ (n = 18), group 3; patients with over 6 metastases in one organ (n = 15), and group 4; patients with 2 or more metastatic organs (n = 77).

**Results:**

It was identified that 49 patients with ED-SCLC had oligometastases (groups 1 + 2) and 92 had polymetastases (groups 3 + 4). The prognoses of patients with ED-SCLC and oligometastases, defined as ≤5 metastases in a single organ, were significantly superior to those of patients with polymetastases [16.0 (95% CI, 11.0–21.0) months vs. 6.9 (95% CI, 6.0–7.8) months; p<0.001]. 43 of 49 patients with ED-SCLC and oligometastases were relapsed after initial chemotherapy, and 38 (88%) experienced local recurrence.

**Conclusions:**

Patients with ED-SCLC and oligometastases may have improved survival than those with polymetastases. As oligometastatic ED-SCLC tends to recur locally, local therapy combined with systemic chemotherapy may be a treatment option.

## Introduction

Lung cancer is the leading cause of cancer mortality worldwide. Small cell lung cancer (SCLC) accounts for 12–15% of all lung cancer cases and is an aggressive disease characterized by widely disseminated metastases and a poor prognosis [1–3], although it is one of the most chemotherapy-sensitive solid tumor types [4–7]. Approximately 60–70% of patients with SCLC present with metastasis beyond a safe radiotherapy field, which is defined as extensive disease (ED). The standard treatment for ED-SCLC is systemic chemotherapy, and the median survival is 8–13 months [5, 6, 8–11].

Oligometastasis is correlated with the management of patients with several solid tumors including non-SCLC. Oligometastasis is defined as having 1–5 metastatic regions; it was shown that patients with oligometastases survive longer than those with polymetastases [12]. The patients with limited metastases such as oligometastases had been aggressively treated with surgical resection and/or radiation therapy [13–16]. International Association for the Study of Lung Cancer proposed the 8th edition of the TNM classification for lung cancer in 2015 [17]. One of the most significant differences between the 7th and 8th editions is the change in the number of M descriptors from three (M0, M1a, and M1b) to four (M0, M1a, M1b, and M1c). It was showed that patients with non-SCLC and single metastasis have a better prognosis than those with polymetastases [17].

Although SCLC is considered a systemic disease, the number of metastatic sites was reported to be associated with the prognosis of patients with ED-SCLC [18, 19]. However, the differences in survival times between oligometastatic and polymetastatic ED-SCLC remain unclear. This retrospective study aimed to analyze whether oligometastases occur in patients with ED-SCLC patients according to the number of metastatic sites at diagnosis, and to examine the efficacy of treatment in these patients.

## Materials and methods

### Study design and population

This retrospective study enrolled 141 consecutive patients with a pathological diagnosed of SCLC in whom first-line systemic chemotherapy was initiated between January 2008 and December 2016 at the Kitasato University Hospital (Kanagawa, Japan). The results of computed tomography (CT), positron emission tomography (PET), bone scintigraphy, and brain magnetic resonance imaging (MRI) were reviewed to classify the patients using the 8th TNM classification before treatment. Patients with distant metastasis (stage M1a, M1b and M1c) were classified as ED-SCLC.

### Subgroups of ED-SCLC patients

The concept of oligometastasis was first proposed in Hellman’s study [12]; oligometastasis is a state in which cancer patients have 1–5 metastatic sites. In this study, ED-SCLC patients were divided into four subgroups: group 1, patients with solitary metastatic site in one organ; group 2, patients with 2–5 metastatic sites in one organ; group 3, patients with over 6 metastases in one organ; and group 4, patients with 2 or more metastatic organs.

### Collection of clinical data

For each patient, the following data were extracted in addition to staging: age at diagnosis, sex, smoking status, the Eastern Cooperative Oncology Group performance status (PS), and laboratory data [including levels of albumin, lactate dehydrogenase (LDH), alkaline phosphatase, sodium, neuron-specific enolase, and pro-gastrin-releasing peptide] obtained before initial chemotherapy. We examined the efficacy of cytotoxic chemotherapy and the sites of recurrence in oligometastatic and polymetastatic patients with ED-SCLC. Survival was measured from the date of diagnosis of SCLC to the date of treatment failure (death or disease progression) or last date of follow-up (data cut-off: October 31, 2017). Furthermore, we investigated the efficacy of local radiotherapy on patients with ED-SCLC and oligometastases. The tumor response was classified in accordance with the Response Evaluation Criteria for Solid Tumors (version 1.1), based on the patients’ complete medical histories and results of physical examinations, chest radiography, CT of the chest and abdomen, and other procedures such as brain MRI, PET, and bone scintigraphy.

### Statistical analysis

The comparison of survival between the different subgroups was performed using the Kaplan–Meier method. Differences in progression-free survival (PFS) and overall survival (OS) between the subgroups based on prognostic factors were compared using the log-rank test. The Cox proportional hazards model was used for univariate and multivariate analyses. A two-tailed p value <0.05 indicated a significant difference for all analyses. The multivariate analysis was performed after adjusting for covariates that included the significant clinical factors in the univariate analyses. All analyses were performed using the SPSS software, version 25.0 (SPSS, Chicago, Illinois, USA).

### Ethics

This study was approved by the Kitasato University Medical Ethics Organization (B17-253). The need for patient consent was waived owing to the retrospective design of the study.

## Results

### Patient characteristics

The baseline clinical characteristics of the patients are shown in Table 1. 16 of 141 patients (82%) were men, and the median age was 70 (range, 42–92) years. A total of 64 patients (45%) had single organ metastasis, whereas 77 (55%) had multiple organ metastases. All 141 patients underwent chest and abdominal CT scans, and 106 (75%), 23 (16%), and 137 (97%) patients underwent PET, bone scintigraphy, and MRI or CT of the brain as part of the initial staging evaluation, respectively. The single organ metastatic sites were the brain (n = 6), lung (n = 3), liver (n = 16), adrenal gland (n = 6), bone (n = 22), and other sites [n = 11; lymph node (n = 9), thyroid (n = 1), and pancreas (n = 1)].

**Table 1:** Patient characteristics in this study (n = 141).

	Group 1	Group 2	Group 3	Group 4
	(n = 31)	(n = 18)	(n = 15)	(n = 77)
**Age**, n				
Median (range), years	71 (49–82)	68 (49–78)	73 (51–92)	70 (42–89)
<75 years	25	14	8	54
≥75 years	6	4	7	23
**Sex**, n				
Male	27	14	12	63
Female	4	4	3	14
**Smoking status**, n				
Never	1	1	2	3
Former/current	29	17	12	73
Unknown	1	0	1	1
**ECOG PS**, n				
0/1	25	13	9	40
2/3/4	6	5	6	37
**Metastatic sites**, n				
Brain	5	1	0	-
Lung	1	1	1	-
Liver	5	5	6	-
Adrenal gland	5	1	0	-
Bone	6	8	8	-
Others	9	2	0	-
**Blood tests**, mean ± SD				
Albumin, g/dL	3.8 ± 0.4	3.6 ± 0.5	3.5 ± 0.6	3.6 ± 0.4
LDH, IU/L	252.3 ± 98.0	394.7 ± 315.0	473.3 ± 347.6	594.8 ± 1015.7
ALP, U/L	275.9± 88.8	339.5 ± 232.0	403.8 ± 260.3	421.6 ± 317.1
Sodium, mEq/L	139.1 ± 3.6	137.5 ± 5.7	137.9 ± 4.4	136.5 ± 5.9
NSE, ng/mL	46.5 ± 54.4	123.3 ± 195.7	132.8 ± 137.2	102.8 ± 119.4
Pro-GRP, ng/mL	1,627.4 ± 3,577.4	1,426.3 ± 1,773.1	2,320.9 ± 3,670.4	3,798.6±12,424.2

**Note:** ECOG, Eastern Cooperative Oncology Group; PS, performance status; SD, standard deviation; LDH, lactate dehydrogenase; ALP, alkaline phosphatase; NSE, neuron-specific enolase; pro-GRP, pro-gastrin-releasing peptide

### Chemotherapy and radiotherapy for patients with ED-SCLC

All patients underwent systemic chemotherapy. The most commonly used first-line chemotherapy regimens were carboplatin-based doublet therapy in 69 patients (49%) and amrubicin monotherapy in 72 patients (51%). A median of 2 (range, 1–8) regimens were received in this study. The treatment details are presented in Table 2. The 141 patients had an OS of 10.0 [95% confidence interval (CI), 8.4–11.6] months, and PFS of 5.5 (95% CI, 4.8–6.3) months after first-line chemotherapy. The numbers of patients who underwent thoracic radiotherapy (TRT) were 6 of 31 (19%) in group 1, 1 of 18 (6%) in group 2, 3 of 15 (20%) in group 3, and 7 of 77 (9%) in group 4. There was no statistically significant survival difference between the oligometastatic and polymetastatic ED-SCLC patients (p = 0.59).

**Table 2:** Efficacy of Treatment in the ED-SCLC patients (n = 141).

	Group 1	Group 2	Group 3	Group 4
	(n = 31)	(n = 18)	(n = 15)	(n = 77)
**Regimen of initial chemotherapy**, *n* (%)				
CDDP + CPT	3 (9.7)	1 (5.6)	3 (20.0)	8 (10.4)
CDDP + ETP	0 (0)	2 (11.1)	0 (0)	4 (5.2)
CBDCA + CPT	1 (3.2)	1 (5.6)	0 (0)	0 (0)
CBDCA + ETP	12 (38.7)	3 (16.7)	3 (20.0)	19 (24.7)
AMR	11 (35.5)	8 (44.4)	7 (46.7)	36 (46.8)
CPT + AMR	3 (9.7)	1 (5.6)	2 (13.3)	4 (5.2)
Others*	1 (3.2)	2 (11.1)	0 (0)	6 (7.8)
**Number of regimen administered**, n (%)				
1	6 (19.4)	8 (44.4)	6 (40.0)	42 (54.5)
2	12 (38.7)	6 (33.3)	7 (46.7)	23 (29.9)
≥3	13 (41.9)	4 (22.3)	2 (13.3)	12 (15.6)
**Response to initial chemotherapy**, n (%)				
Partial response	24 (77.4)	10 (55.6)	8 (53.3)	49 (63.6)
Stable disease	4 (12.9)	4 (22.2)	4 (26.7)	10 (13.0)
Progressive disease	1 (3.2)	1 (5.6)	3 (20.0)	10 (13.0)
Not evaluated	2 (6.5)	3 (16.7)	0 (0)	8 (10.4)
**Thoracic radiotherapy**, n (%)				
Yes	6 (19.4)	1 (5.6)	3 (16.7)	7 (9.1)
No	25 (80.6)	17 (94.4)	12 (83.3)	70 (90.9)

**Note:** CDDP, cisplatin; CPT, irinotecan; ETP, etoposide; CBDCA, carboplatin; AMR, amrubicin

### Comparison of survival between the subgroups of ED-SCLC patients

**Fig 1:**
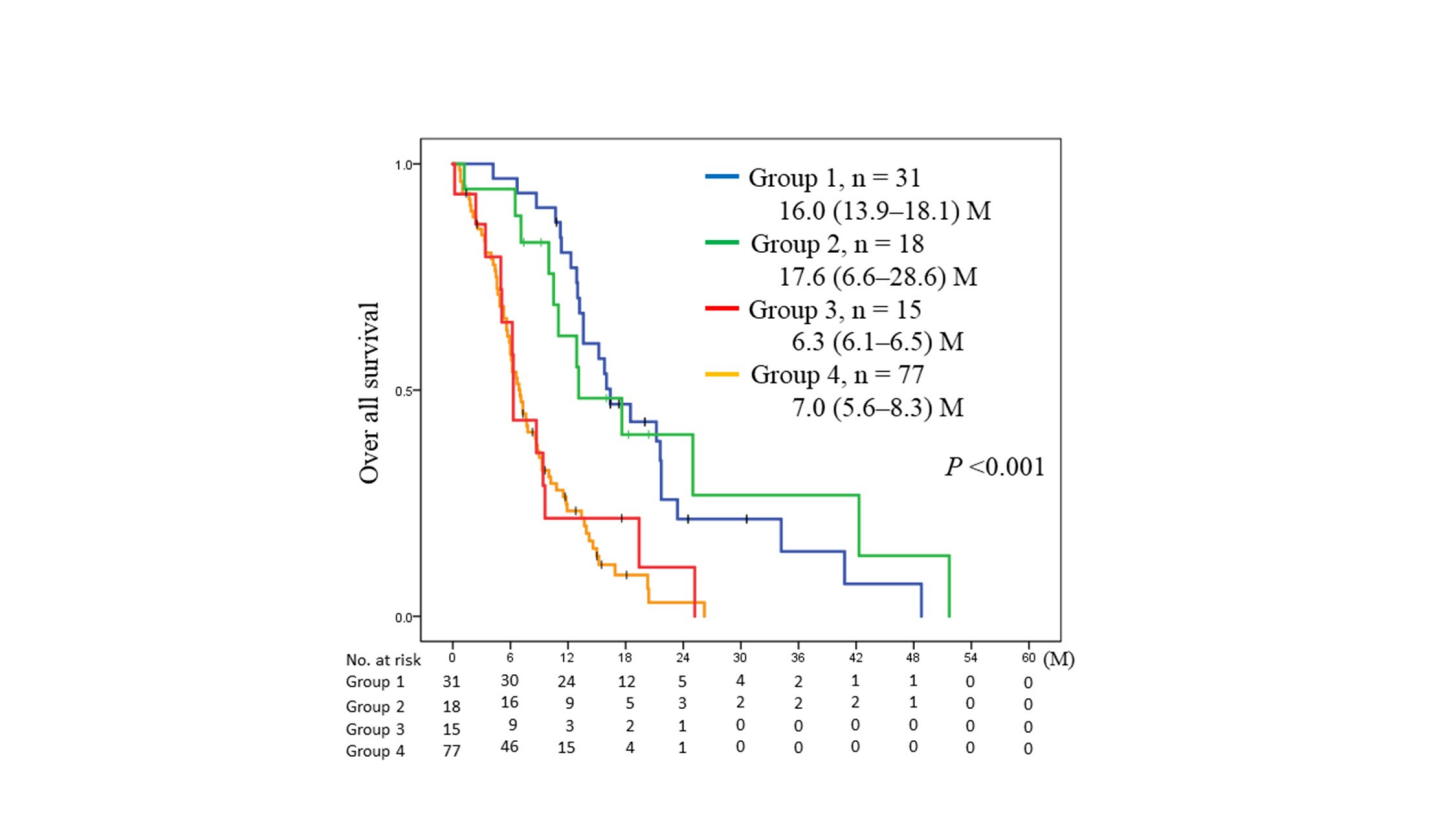
Kaplan–Meier analysis of overall survival (OS) in group 1 (*blue*) vs. group 2 (*green*) vs. group 3 (*red*) vs. group 4 (*yellow*). *P* values were determined using the log-rank test; the number of individuals in each group and median survival [95% confidence interval (CI)] are indicated. Group 1 included patients with a solitary metastatic site in one organ, group 2 patients with 2–5 metastatic sites in one organ, group 3 those with ≥6 metastases in one organ, and group 4 those with ≥2 organs with metastases. M; months.

The OS of patients with ED-SCLC in groups 1, 2, 3, and 4 were 16.0 (95% CI, 13.9–18.1), 17.6 (95% CI, 6.6–28.6), 6.3 (95% CI, 6.1–6.5), and 7.0 (95% CI, 5.6–8.4) months, respectively. Of the entire study cohort, the OS of group 1 was similar to that of group 2 (p = 0.74) and superior to that of group 3 (p = 0.001) and group 4 (p <0.001). Moreover, the OS of group 2 was superior to that of the group 3 (p = 0.011) and group 4 (p = 0.001). The OS of group 3 was similar to that of group 4 (p = 0.82) (Fig 1).

**Fig 2:**
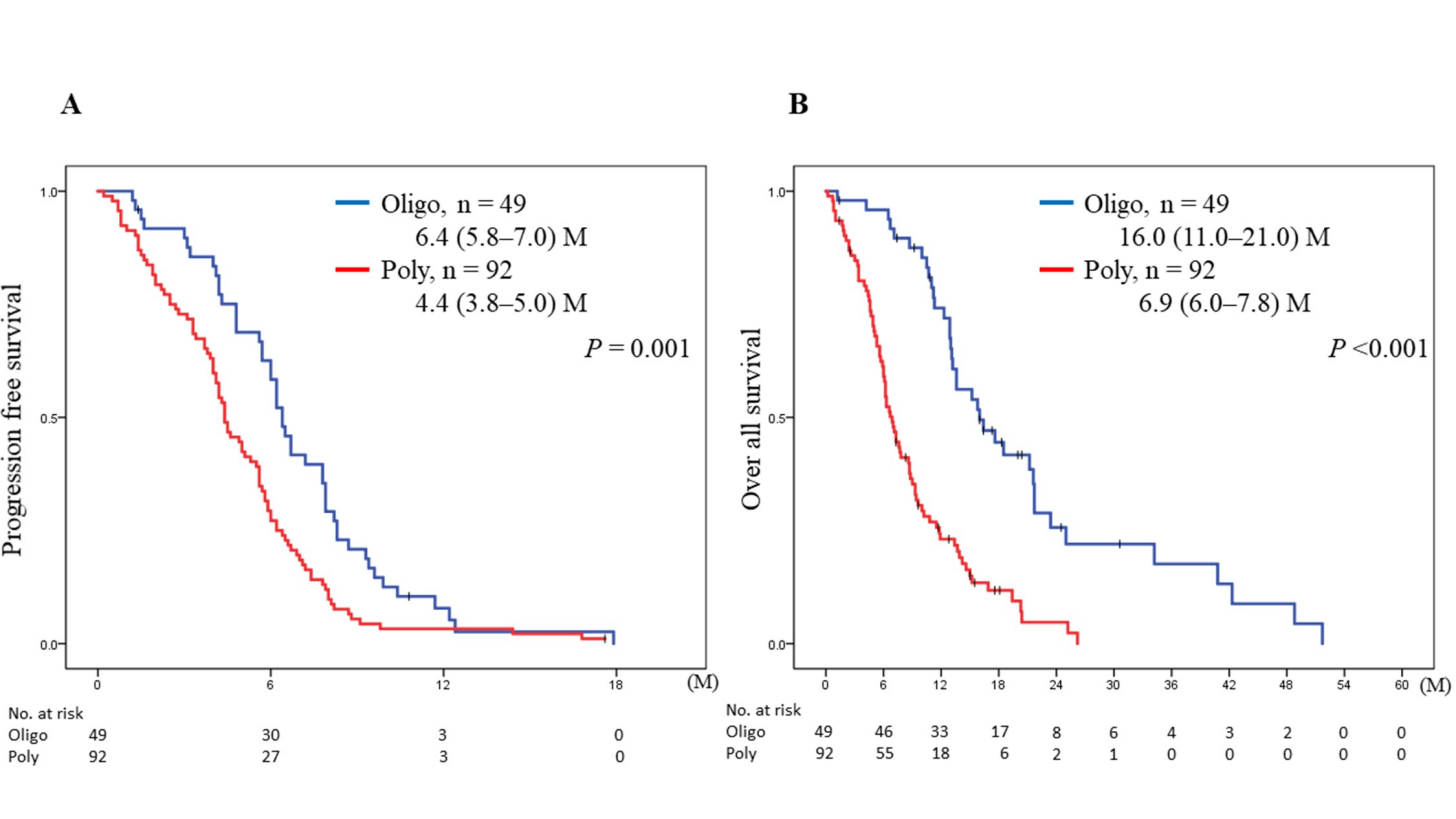
**Kaplan–Meier analyses of progression-free survival (PFS) (A) and overall survival (OS) (B) of patients with oligometastases (*blue*: group 1 and 2) vs. patients with polymetastases (*red*: group 3 and 4) treated with chemotherapy.** P values were determined using the log-rank test; the number of individuals in each group and median survival (95% CI) are indicated. M; months, Oligo; oligometastases, Poly; polymetastases.

The PFS after the first-line chemotherapy was significantly different between patients with 1–5 metastases in one organ (groups 1 and 2; oligometastases) and those with ≥6 metastases in one organ or metastases in multiple organs (groups 3 and 4; polymetastases) [6.4 (95% CI, 5.8–7.0) vs. 4.4 (95% CI, 3.8–5.0) months; p = 0.001; Fig 2A]. The OS of patients with ED-SCLC and oligometastases was also significantly better than that of patients with polymetastases [16.0 (95% CI, 11.0–21.0) vs. 6.9 (95% CI, 6.0–7.8) months; p <0.001; Fig 2B].

In the univariate survival analysis of patients with ED-SCLC who received chemotherapy, patients with polymetastases had an unfavorable prognosis [hazard ratio (HR) 1.83; 95% CI, 1.48–2.27; p <0.001] in addition to those with known prognostic factors such as older age, lower PS, and serum levels of LDH and sodium. In the multivariate analysis, having polymetastases was significantly associated with an unfavorable prognosis when compared to having oligometastases (HR 1.76; 95% CI, 1.41–2.21; p = <0.001) (Table 3).

**Table 3:** Univariate and multivariate analyses for overall survival in the ED-SCLC patients (n = 141).

	Univariate			Multivariate		
	HR	95% CI	*P*	HR	95% CI	*P*
Age	1.83	1.23–2.74	0.003	1.89	1.25–2.86	0.003
≥75 vs. <75						
Sex	1.09	0.68–1.74	0.72			
Female vs. male						
Smoking status	1.30	0.53–3.20	0.57			
Former/Current vs. never						
ECOG PS	1.62	1.11–2.37	0.01	1.31	0.88–1.95	0.18
2–4 vs. 0–1						
Pleural effusion	1.00	0.69–1.45	1.00			
Yes vs. no						
Albumin	1.23	0.78–1.93	0.37			
Low vs. normal						
LDH	2.14	1.34–3.42	0.002	1.56	0.96–2.54	0.076
High vs. normal						
ALP	1.36	0.94–1.97	0.10			
High vs. low						
Sodium	1.88	1.28–2.78	0.001	1.65	1.11–2.44	0.013
Low vs. normal						
Platinum-based chemotherapy	0.71	0.49–1.02	0.03			
Yes vs. No						
Metastases	1.83	1.48–2.27	<0.001	1.76	1.41–2.21	<0.001
Oligo- vs. polymetastases						

**Note:** HR, hazard ratio; CI, confidence interval; ECOG, Eastern Cooperative Oncology Group; PS, performance status; LDH, lactate dehydrogenase; ALP, alkaline phosphatase

### Patterns of progression in oligometastatic ED-SCLC

**Fig 3:**
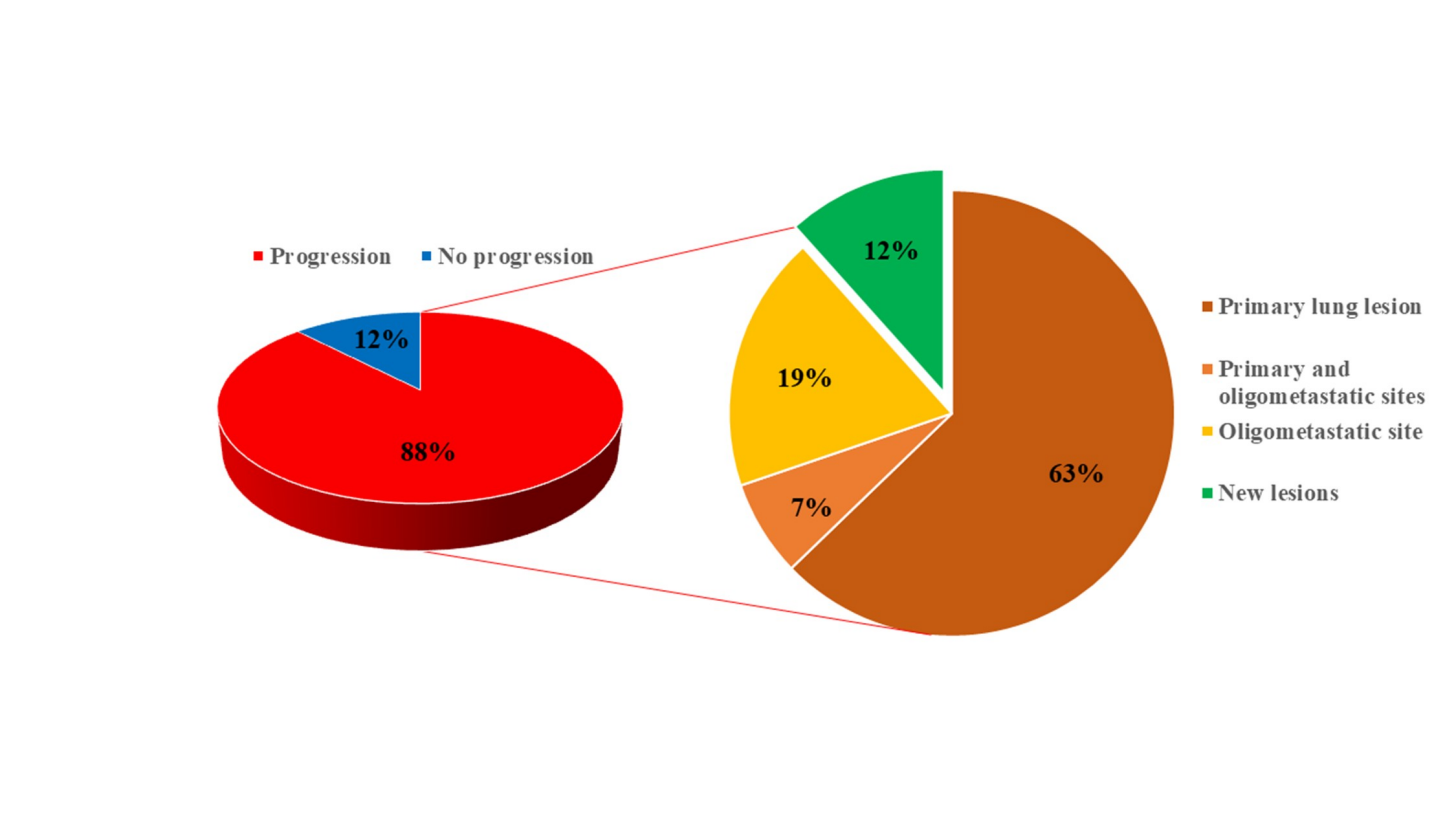
Pattern of recurrence in the oligometastatic ED-SCLC patients (n = 49). 43 of 49 (88%) ED-SCLC patients had relapsed after initial chemotherapy. Of the patients who relapsed, 27 (63%) exacerbated primary lung lesion (*red*), 8 (19%) relapsed at oligometastatic sites (*orange*) and 3 (7%) relapsed in both the primary and the oligometastatic sites (*light orange*). 5 (12%) patients had recurrence into new lesions (*green*).

We then investigated the patterns of recurrence in patients with ED-SCLC and oligometastases. In 43 of 49 (88%) patients with oligometastases, relapses occurred in the primary lung tumor in 27 (63%) patients, metastatic site in 8 (19%) patients and 3 (7%) patients experiencing both (Fig 3). In 35 of 43 (81%) patients, the primary tumor had progressed. In all patients with brain, lung, or adrenal gland metastases, relapse occurred in both the primary tumor and metastatic organs before chemotherapy.

Next, we evaluated the efficacy of local radiotherapy in patients with oligometastatic ED-SCLC. Patients with oligometastatic ED-SCLC who underwent thoracic radiotherapy (TRT; n = 7) tended to have better survival time than those who did not underwent TRT (n = 42), although there was no significant difference [23.4 (95% CI, 10.1–36.7) months vs. 15.8 (95% CI, 12.7–18.9) months; p = 0.42; S1 Fig].

## Discussion

This study suggests that oligometastatic ED-SCLC has a better prognosis than polymetastatic disease and is characterized by local recurrence. Some studies evaluated the revised M descriptors of the 8th edition of TNM classification in patients with ED-SCLC [20, 21]. In this study, the OS of patients with ED-SCLC and a single metastasis in one organ (group 1), categorized as the M1b stage based on the 8th edition of TNM classification, was 16.0 (95% CI, 11.4–21.0) months. Furthermore, the OS of patients with ED-SCLC and 2–5 metastases in one organ (group 2) was not inferior to that of group 1 patients and was superior to that of patients with ≥6 metastases in one organ (group 3) or ≥2 metastatic organs (group 4). Oligometastases means that patients have a limited number of metastases and organ site(s) and may have a more indolent biology and progression at existing sites without widespread metastases. These patients have a better prognosis than those who have widespread metastases [12]. Based on the concept of oligometastasis, patients with ED-SCLC and 1–5 metastases in one organ were defined as having “oligometastatic” ED-SCLC.

In this study, we observed a significant difference in the outcomes of patients with ED-SCLC patients and oligometastases depending on the site of oligometastatic organs (S2 Fig). The survival of patients with oligometastases in the brain [n = 6; 40.8 (95% CI, 10.6–71.0) months] or adrenal gland [n = 6; 34.2 (95% CI, 3.4–65.0) months] metastases was better than that of those with metastases in other organs [n = 37; 13.6 (95% CI, 10.7–16.5) months]. It was reported that only 27% of patients with asymptomatic brain metastases responded to systemic chemotherapy [22]. Four of six patients (67%) with brain metastases had recurrence in the brain after initial chemotherapy. Among patients with non-SCLC, the prognosis of single brain metastasis has been shown to be considerably better than that of multiple brain metastases; furthermore, patients with non-SCLC and single brain metastasis benefit from locally aggressive and ablative treatments such as stereotactic irradiation or surgery [23–25]. Local radiation therapy for brain metastases is also considered to be effective in SCLC patients [26]. In this study, 6 patients with ≤5 brain metastases underwent local treatments; one patient underwent brain tumor excision before initiating chemotherapy, all patients underwent whole-brain radiation therapy, and 3 of 6 patients underwent a cyber knife treatment for exacerbation of brain metastases after WBRT. Brain irradiation underwent somewhere during treatment after chemotherapy. This finding suggests that local brain irradiation may be beneficial in patients with systemic disease in ED-SCLC patients with oligometastases.

Regarding the occurrence of oligometastases in adrenal glands, initial staging should be carefully performed as the presence of a tumor does not necessarily represent metastasis but could indicate a benign adenoma (2–9% of cases) [27]. Although CT, PET, and MRI are useful tools for the detection of adrenal metastases, it should be noted these non-invasive modalities have some limitations [28–33]. For distinguishing adrenal metastases, PET-CT with a mean attenuation ≥10 HU and SUV max ≥3.1 had 97.3% sensitivity and 86.2% specificity [34]. Six patients with adrenal metastasis in this study met the condition of PET-CT. Regarding the cause of death of the six patients with adrenal metastases, three had exacerbations of the primary tumor, one had brain metastases, and the remaining had disease progression while managing adverse events, febrile neutropenia, and renal failure due to chemotherapy. None of the patients received treatment for local adrenal metastasis, and none died from adrenal metastasis.

In previous studies, 75–90% of patients with ED-SCLC had residual intrathoracic disease, 90% developed intrathoracic progression after chemotherapy [35], and most patients with ED-SCLC died of thoracic progression and associated complications [36]. In this study, of the 38 patients with 1–5 metastases (excluding those who survived or whose cause of death was unknown), 27 (71%) died due to exacerbation of the primary lesions. The patients with oligometastatic ED-SCLC who underwent TRT (n = 7) trended to have a better prognosis than those who did not undergo TRT (n = 42). A recent study showed that the introduction of high-dose radiation therapy to chemotherapy was an effective treatment strategy for patients with ED-SCLC as it significantly improved patients’ survival [37]. Furthermore, patients with 1–4 extracranial metastases who underwent consolidative radiation therapy had a 50% reduction in intrathoracic recurrence than those who did not (80% vs. 44%, respectively; p = 0.001) [38]. In a previous report, TRT in ED-SCLC patients with 0–2 distant organ metastases significantly improved PFS (n = 61; HR = 2.02; p = 0.003) [39]. Thus, we suggested that patients with oligometastatic ED-SCLC, who had mainly intrathoracic progression, should be indicated for intrathoracic irradiation.

Our study had several limitations. This study was a retrospective study conducted at a single institution; therefore, our results cannot be regarded as definitive. The sample size may not have been sufficient. Some of the patients received local radiotherapy, but others did not. Patients who received high- or low-dose radiotherapy and the period when they received the treatment were not identified. To determine which patients will most likely benefit from escalated doses of radiotherapy, a large prospective study is needed to corroborate our findings.

## Conclusion

Patients with oligometastatic ED-SCLC may have a better prognosis than those with polymetastatic disease. Patients with oligometastatic ED-SCLC patients tend to experience local recurrence. For oligo- and polymetastatic ED-SCLC patients, treatment strategies should be developed separately. Local treatment combined with systemic chemotherapy may be a treatment option for the oligometastatic ED-SCLC patients, especially in patients with cerebral or adrenal grand oligometastases.

## Supporting information

S1 Fig Kaplan–Meier analysis of overall survival (OS) for the patients treated with thoracic radiotherapy (TRT). Kaplan–Meier analysis of OS for the patients with oligometastases (*blue*) vs. the patients with polymetastases (*red*) treated with TRT. *P* values were determined using the log-rank test; the number of individuals in each group and median survival (95% confidence interval) are indicated. M; months.
(TIF)

S2 Fig Kaplan–Meier analysis of overall survival (OS) in patients with oligometastases for each metastatic organ. Kaplan–Meier analysis of OS in patients with oligometastases for each oligometastatic site: adrenal gland (*blue*), brain (*green*), liver (black), lung (*yellow*), bone metastases (*gray*), the others (*red*). The number of individuals in each group and median survival (95% confidence interval) are indicated. M; months.
(TIF)

S1 Table Patient characteristics and treatment outcome. (XLSX)
