# Supplementary figures and images for: Prognostic differences between oligometastatic and polymetastatic extensive disease-small cell lung cancer
Source: PLoS One. 2019 Apr 19;14(4):e0214599. doi: 10.1371/journal.pone.0214599 (PMC6474590; doi:10.1371/journal.pone.0214599)

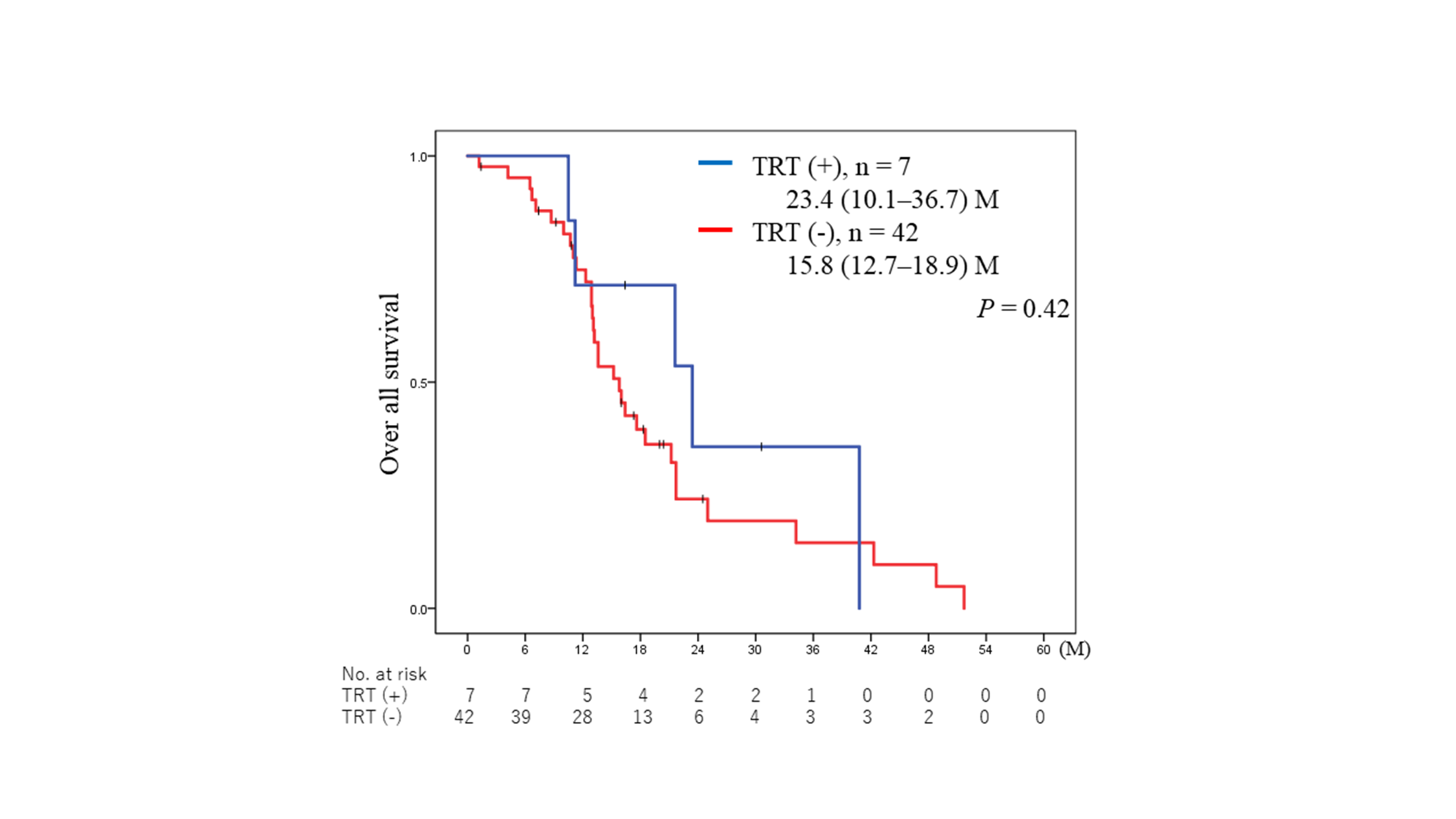

Supplement: S1 Fig — Kaplan–Meier analysis of OS for the patients with oligometastases (blue) vs. the patients with polymetastases (red) treated with TRT. P values were determined using the log-rank test; the number of individuals in each group and median survival (95% confidence interval) are indicated. M; months. (TIF) [file pone.0214599.s001.tif]

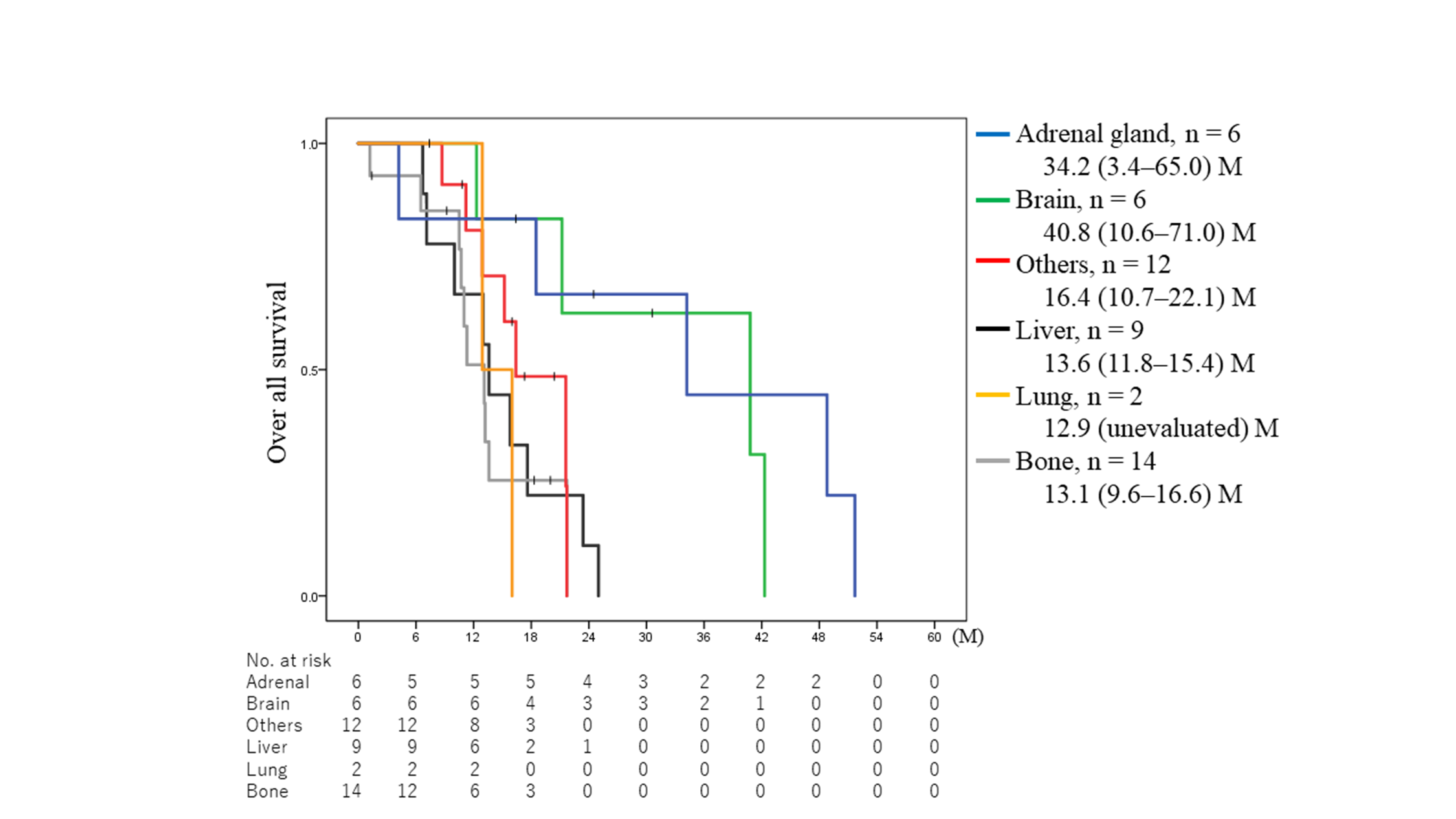

Supplement: S2 Fig — Kaplan–Meier analysis of OS in patients with oligometastases for each oligometastatic site: adrenal gland (blue), brain (green), liver (black), lung (yellow), bone metastases (gray), the others (red). The number of individuals in each group and median survival (95% confidence interval) are indicated. M; months. (TIF) [file pone.0214599.s002.tif]
